# Genome-Wide Identification of the SPP/SPPL Gene Family and *BnaSPPL4* Regulating Male Fertility in Rapeseed (*Brassica napus* L.)

**DOI:** 10.3390/ijms25073936

**Published:** 2024-04-01

**Authors:** Guangze Li, Wenjun Zhu, Minyu Tian, Rong Liu, Ying Ruan, Chunlin Liu

**Affiliations:** 1 Yuelushan Laboratory, Hunan Agricultural University, Changsha 410128, China; 15379295903@163.com (G.L.); 17809972112@163.com (W.Z.); tianminyu1995@126.com (M.T.); rrailyf@163.com (R.L.); yingruan@hotmail.com (Y.R.); 2 Key Laboratory of Hunan Provincial on Crop Epigenetic Regulation and Development, Hunan Agricultural University, Changsha 410128, China

**Keywords:** *Brassica napus* L., *SPP/SPPL* gene family, expression profile, pollen, male sterility

## Abstract

Signal peptide peptidase (SPP) and its homologs, signal peptide peptidase-like (SPPL) proteases, are members of the GxGD-type aspartyl protease family, which is widespread in plants and animals and is a class of transmembrane proteins with significant biological functions. *SPP/SPPLs* have been identified; however, the functions of SPP/SPPL in rapeseed (*Brassica napus* L.) have not been reported. In this study, 26 *SPP/SPPLs* were identified in rapeseed and categorized into three groups: SPP, SPPL2, and SPPL3. These members mainly contained the Peptidase_A22 and PA domains, which were distributed on 17 out of 19 chromosomes. Evolutionary analyses indicated that *BnaSPP/SPPLs* evolved with a large number of whole-genome duplication (WGD) events and strong purifying selection. Members are widely expressed and play a key role in the growth and development of rapeseed. The regulation of rapeseed pollen fertility by the *BnaSPPL4* gene was further validated through experiments based on bioinformatics analysis, concluding that *BnaSPPL4* silencing causes male sterility. Cytological observation showed that male infertility caused by loss of *BnaSPPL4* gene function occurs late in the mononucleate stage due to microspore dysplasia.

## 1. Introduction

Signal peptide peptidase (SPP) and its homologs, signal peptide peptidase-like (SPPL) proteases, were first discovered in the human genome [1,2,3]. The *SPP/SPPL* family [4], along with the presenilin (Ps) family [5], make up aspartyl intramembrane-cleaving proteases (I-CLiPs) based on their role in cleaving proteases in the endoplasmic reticulum. SPP/SPPL proteins can be found in different eukaryotic organisms [1,2,3]. There are six members of the *SPP/SPPL* family known to exist in mammals: SPP, SPPL2 (SPPL2a, SPPL2b, and SPPL2c), and SPPL3 [6]. Fish only has one member of SPPL2 [7], whereas drosophila has two members that resemble human SPPL3 and SPP [8]. Like human SPP, the plasmodium genome encodes a single member [9]. Similar SPP/SPPL sequences have also been discovered in Arabidopsis and rice (*Oryza sativa* L.) [1,3]. Six *SPP/SPPLs* have been designated *ATSPP*, *ATSPPL1*, *ATSPPL2*, *ATSPPL3*, *ATSPPL4*, and *ATSPPL5* in *Arabidopsis thaliana*. Through the construction of a phylogenetic tree, these genes were classified into three subgroups based on their distribution in *A. thaliana*, humans, zebrafish, and mice: SPP, SPPL2, and SPPL3. *ATSPP* is a part of the SPP subgroup; *ATSPPL1* is a member of the SPPL3 subgroup; and *ATSPPL2*, *ATSPPL3*, *ATSPPL4*, and *ATSPPL5* belong to the SPPL2 subgroup [10].

Intramembrane proteases mainly hydrolyze their protein substrates using catalytic centers located on the surface of the membrane or across the membrane segment. The catalytic centers of SPP/SPPL proteins are composed of Y/FD, GxGD, and QPALLY [11]. Nine transmembrane domain (TMD) topologies have been predicted based on similarity to Ps, and the hydrophilic loop luminal orientation between TMD 6 and TMD 7, as well as the luminal and cytosolic orientation of the N- and C-termini, respectively, have been verified [11]. The Y/FD and GxGD motifs are present in TMD 6 and TMD 7, respectively [1,3]. However, in contrast to Ps, the overall topology of SPP/SPPL is inverted [11]. Furthermore, all *SPP/SPPLs* include a conserved QPALLY motif in the N-terminal region of TMD 9, in addition to the motifs in TMD 6 and TMD 7 [1,2,3]. Ps undergoes self-protein hydrolysis and is activated upon binding to polymer complexes [12,13]. Conversely, the overall structure of SPP/SPPL proteins and the absence of conserved hydrophobic amino acid sequences allow them to function without the need for hydrolysis of other proteins [11,14].

The biological functions of proteases are determined by their substrates. Hundreds of substrates, including Notch receptors and amyloid precursor proteins, can be cleaved by Ps [15,16], but display significant selective type I membrane protein cleavage [4]. Since the TMDs of SPP/SPPL proteins are opposite those of Ps and their N-terminus is found in the extracellular region of the plasma membrane or the lumen of organelles, type II transmembrane protein substrates with an N-terminus located in the cytoplasm are the only substrates recognized by the *SPP/SPPL* family. Numerous substrates for *SPP/SPPL* family members have been found, including the protein substrates Xbp1u [17] and SrbA (*A. nidulans*) [18] of SPP; among the protein substrates of SPPL2a are TNF [19,20] and CD74 [21,22,23,24]; ITM2B [25] and TNF [19,20] are protein substrates of SPPL2b; FVenv [26] is one of the protein substrates of SPPL3, along with GnT-V [27] and HS6ST1 [26]; and different subcellular localizations exhibit different functions in plants and animals. SPP, SPPL2a, SPPL2b, SPPL2c, and SPPL3 were confirmed to be located in the endoplasmic reticulum, lysosome, plasma membrane, and Golgi apparatus, respectively [28]. In zebrafish, the degradation of SPP, SPPL2a, and SPPL3 leads to tail vein elongation, nervous system cell death, and embryonic cell death [7]. Depletion of SPPL2c in mice caused changes to the Golgi apparatus in spermatozoons and the glycosylation pattern of mature spermatozoa, which in turn caused some spermatozoa to die and the motility of mature spermatozoa to decrease [29,30]. Significant male gametophyte abnormalities were seen in heterozygotes produced through T-DNA insertion of ATSPP in *A. thaliana*, suggesting that ATSPP is essential for pollen formation [31]. Although there are many reports on *SPP/SPPL* proteases, their functions in plants have not been extensively studied.

Rapeseed is one of the most important oil crops in the world and provides an important source of edible oil and biofuel [32]; improving its yield is the long-term goal of breeding. Male sterile plants are necessary breeding materials for cross-breeding. The main feature of these plants is abnormal development of the stamen, which cannot produce pollen with a normal function; however, their pistil development is normal, and it can accept normal pollen and be fertilized. There are three main categories of male sterility: cytoplasmic male sterility (CMS), genic male sterility (GMS), and ecological male sterility (EMS). EMS can be divided into photoperiod-sensitive genic male sterility (PGMS) and temperature-sensitive genic male sterility (TGMS) [33,34]. Although many male sterile lines have been obtained through hybridization and natural mutation, the creation of new male sterile lines through genome editing is still one of the most important breeding strategies.

In this study, members of the *SPP/SPPL* family were systematically identified using the genome of *B. napus*. The gene structure, motifs, expression patterns, and physical and chemical properties were examined using bioinformatics tools. At the same time, we successfully constructed the RNA interference (RNAi) expression vector of *BnaSPPL4* and obtained transgenic rapeseeds, and found that the *Bnasppl4* mutant showed obvious male sterility characteristics. Therefore, the reason for male sterility was analyzed through paraffin sectioning.

## 2. Results

### 2.1. Identification and Characterization of SPP/SPPL Family Members

Simple HMM Search in TBtools and the BLASTP program were used to identify members of the *SPP/SPPL* family. A total of 57 members were found in the four species after duplicate sequences were removed and the structure was verified using the NCBI website. Of these, 6 were found in *A. thaliana*, 26 in *B. napus*, 12 in *Brassica rapa*, and 13 in *Brassica oleracea*. The genes were named based on chromosome position information (Supplementary Table S2). The average length of the *BnaSPP/SPPLs* was 393 aa, with the longest length being 541 aa (*BnaSPPL4-2*) and the shortest length being 86 aa (*BnaSPPL3-1*). According to an analysis of the physicochemical properties of *BnaSPP/SPPLs* using Expasy and WoLF PSORT, the proteins were expected to have relative molecular weights between 9.31 kDa (*BnaSPPL3-1*) and 62.51 kDa (*BnaSPPL4-2*). With the theoretical pI ranging from 4.96 to 9.15, nine proteins had a pI < 7 and 17 proteins had a pI > 7. Proteins are classified as unstable if their instability index is more than 40 and stable if it is less than 40. Among the *BnaSPP/SPPLs*, the instability index varied between 26.01 and 48.29. Nine proteins had an instability index exceeding 40, whereas seventeen proteins had an index below 40. The grand average of hydropathicity (GRAVY) of the *BnaSPP/SPPLs* was positive, indicating that they were all hydrophilic proteins. Subcellular localization predictions showed that 26 genes were localized to the plasma membrane, *BnaSPPL1-4* was localized to the vacuole and vesicles, and *BnaSPPL1-6* was localized to the extracell (Table 1).

### 2.2. Phylogenetic and Gene Structure Characterization of BnaSPP/SPPLs

**Figure 1:**
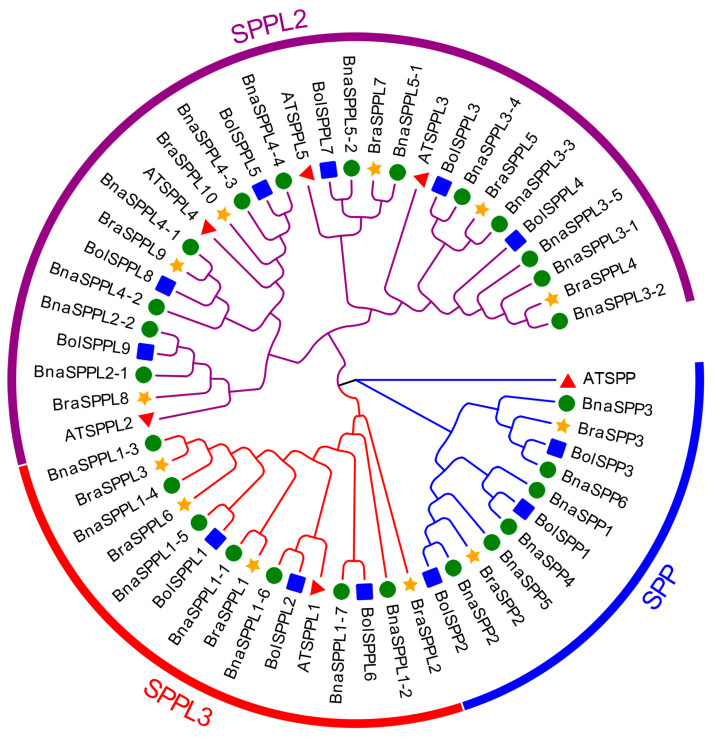
Phylogenetic analysis of *SPP/SPPL* family in *B. napus*, *B. oleracea*, *B. rapa*, and *A. thaliana*, classifying 57 *SPP/SPPLs* into three subgroups: SPP, SPPL2, and SPPL3. Red triangles represent 6 *SPP/SPPLs* in Arabidopsis, green circles represent 26 *SPP/SPPLs* in *B. napus*, blue boxes represent 12 *SPP/SPPLs* in *B. oleracea*, and orange pentagrams represent 13 *SPP/SPPLs* in *B. rapa*.

**Figure 2:**
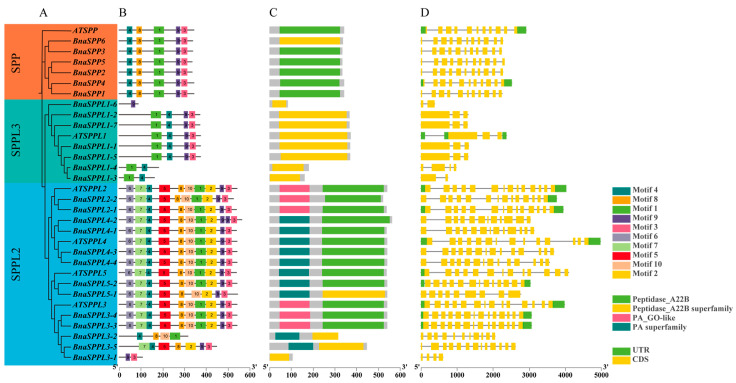
Analysis of gene structure, motif, and domains of *BnaSPP/SPPLs* and *ATSPP/SPPLs*: (**A**) phylogenetic tree; (**B**) conserved motifs; (**C**) domains; (**D**) introns, exons, and UTRs, where introns are marked with black horizontal lines.

We classified 57 members into three subgroups, SPP, SPPL2, and SPPL3, based on the amino acid sequences of *SPP/SPPL* family members from *B. napus*, *B. oleracea*, *B. rapa*, and *A. thaliana*, and using MUSCLE comparisons in MEGA 11.0. There were 12 individuals in the SPP subgroup, 15 in the SPPL3 subgroup, and 30 in the SPPL2 subgroup (Figure 1). MEME and TBtools were used to evaluate the gene structure, conserved motifs, and domains. The results indicated that the highly conserved motif 1, motif 3, motif 4, motif 8, and motif 9 were present in every member of the SPP subgroup. Ten conserved motifs were shared by the majority of members in the SPPL2 subgroup. Nevertheless, *BnaSPPL3-1* only displayed motif 3 and motif 9; *BnaSPPL3-2* only displayed motif 1, motif 4, motif 8, and motif 10; and *BnaSPPL3-5* lacked motif 1, motif 6, and motif 10. In contrast, members of the SPPL3 subgroup primarily expressed motif 1, motif 3, motif 4, and motif 9. *BnaSPPL1-3* and *BnaSPPL1-4* exclusively contained motif 1 and motif 4, whereas *BnaSPPL1-6* only contained motif 9. Gene structure analysis revealed that all members of the SPP subgroup contain eleven exons. Within the SPPL3 subgroup, five members had three exons, while *BnaSPPL1-6* and *BnaSPPL1-3* had two and *BnaSPPL1-4* had four. There were 14 members of the SPPL2 subgroup, all of which contained fourteen exons. *BnaSPPL3-1* had four exons, *BnaSPPL3-2* had eleven, and *BnaSPPL3-5* had thirteen. Members of the *SPP/SPPL* family consist of two main structural domains: the PA domain and the Peptidase_A22B domain. Both the SPP and SPPL3 subgroups contain only the Peptidase_A22B domain, whereas the majority of the members in the SPPL2 subgroup have one PA domain and one Peptidase_A22B domain. However, *BnaSPPL3-1* contains only the Peptidase_A22B domain (Figure 2).

### 2.3. Chromosomal Localization and Collinearity Analysis of BnaSPP/SPPLs

**Figure 3:**
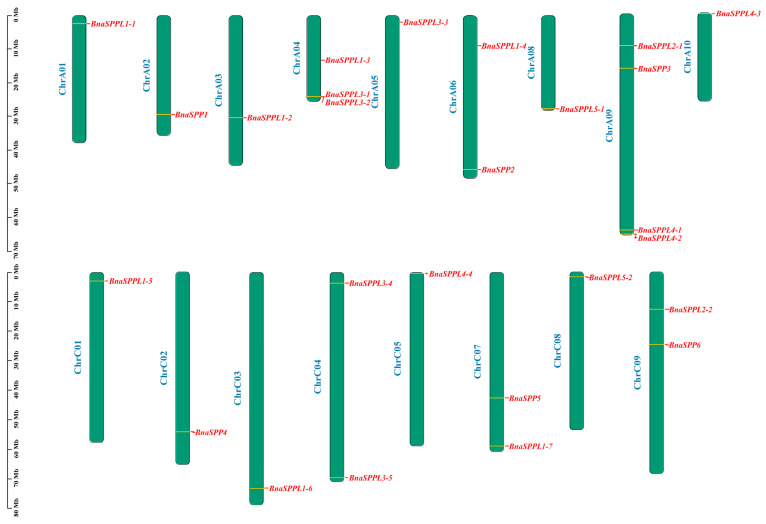
The 26 *BnaSPP/SPPLs* are located on the chromosomes; the length of each chromosome is shown in Mb, the chromosomal number is shown on the left side of the chromosome, and the *BnaSPP/SPPLs* are highlighted in red.

**Figure 4:**
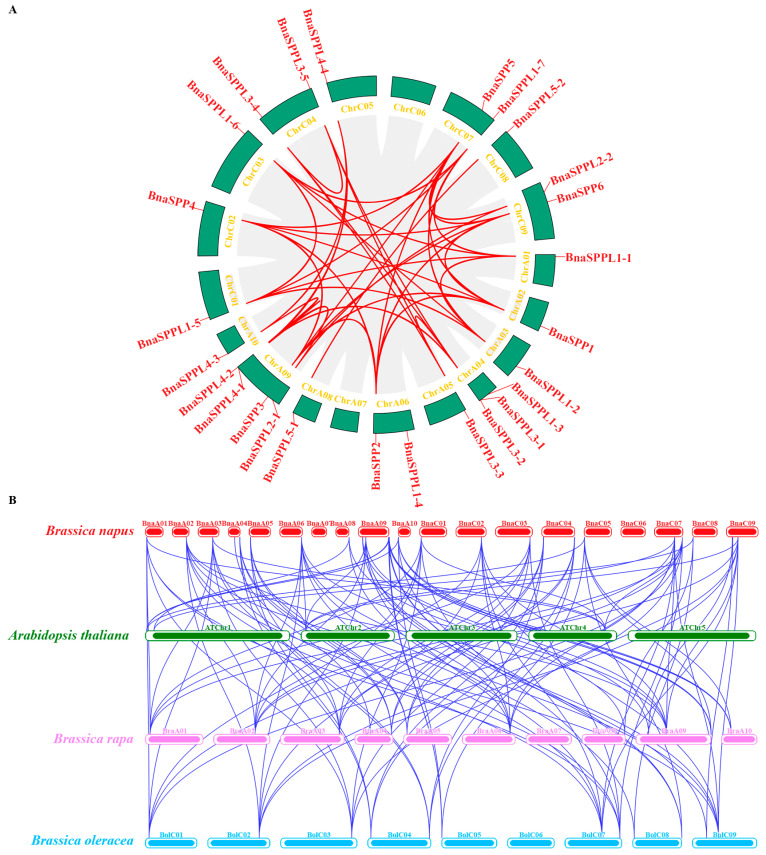
Circular representations of *BnaSPP/SPPL* chromosomal dispersal and inter-chromosomal interactions, as well as synteny analysis of *SPP/SPPL* genes in *B. rapa*, *B. oleracea*, *B. napus*, and Arabidopsis. (**A**) Analysis of syntenic relationships between *BnaSPP/SPPL* paralog pairs; within the schematic image, duplicate pairs of *BnaSPP/SPPLs* are shown by red lines. (**B**) Analysis of syntenic relationships between *BraSPP/SPPLs*, *BolSPP/SPPs*, *BnaSPP/SPPs*, and *ATSPP/SPPs*, as shown by gray lines.

**Figure 5:**
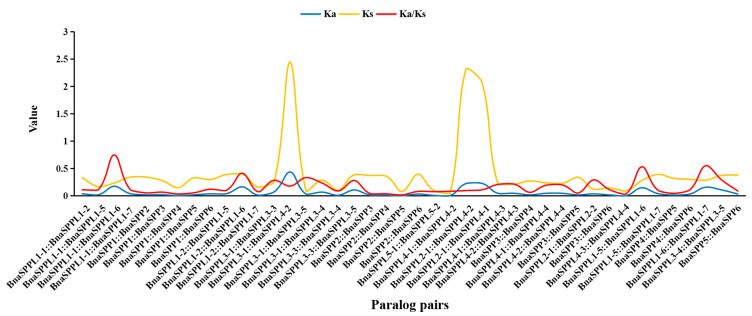
Ka/Ks values of *BnaSPP/SPPL* paralog pairs. Blue lines are Ka values, yellow lines are Ks values, and red lines are Ka/Ks. The X-axis and Y-axis indicate paralog pairs and Ka, Ks, and Ka/Ks values, respectively.

A total of 26 *BnaSPP/SPPLs* were dispersed among 17 chromosomes, with the exception of chromosomes ChrA06 and ChrA07. Of these, only one was found on chromosomes ChrA01, ChrA02, ChrA03, ChrA05, ChrA08, ChrA10, ChrC01, ChrC02, ChrC03, ChrC05, and ChrC08; chromosomes ChrA06, ChrC04, ChrC07, and ChrC09 contained two *BnaSPP/SPPLs*; chromosome ChrA04 contained three; and chromosome ChrA09 contained four (Figure 3). BLAST and MCScanX were used to identify the gene duplication events of *BnaSPP/SPPLs*, which were displayed using Advance Circle (Figure 4A). Numerous WGD events were observed, including a pair of tandem duplicates on chromosome ChrA09 (*BnaSPPL4-1::BnaSPPL4-2*). Along with this, 43 pairs of homologous genes were found; 11 paralog pairs came from the genomes of group A, 8 came from the genome of C, and 24 came from WGD in the genomes of groups A and C. In order to assess the evolutionary mode of *BnaSPP/SPPLs*, the Ka/Ks of 43 homologous gene pairs was computed. The findings indicated that the Ka/Ks of 42 homologous genes was less than 1. The only exception to this was *BnaSPP5::BnaSPPL2-2*, for which the Ks value could not be computed owing to its high degree of differentiation. Therefore, the evolutionary mode was purifying selection (Figure 5, Supplementary Table S3). A collinear events analysis of *A. thaliana*, *B. napus*, *B. oleracea*, and *B. rapa* was carried out in this study in order to analyze the evolutionary mechanism of *SPP/SPPL* family members in different species. The results showed that 15 paralog pairs were formed between *B. napus* and *A. thaliana*, 49 pairs between *B. napus* and *B. rapa*, and 53 pairs between *B. napus* and *B. oleracea* (Figure 4B).

### 2.4. Analyzing the Expression Pattern of BnaSPP/SPPLs in Different Tissues

**Figure 6:**
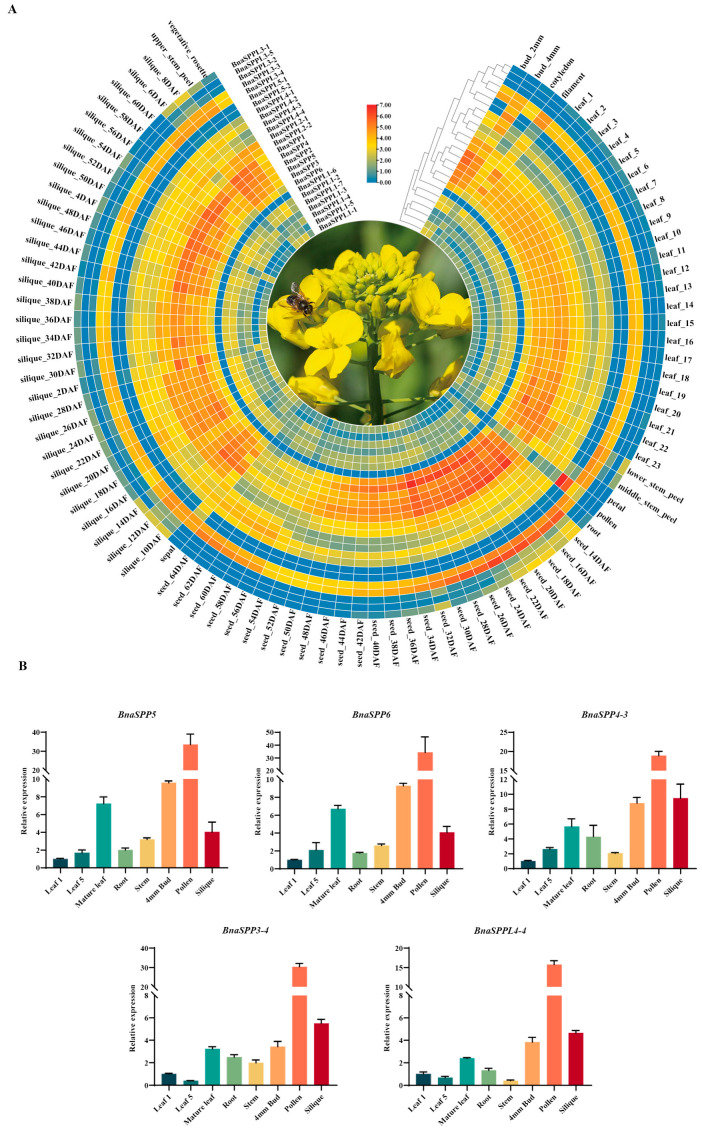
Expression pattern analysis of *BnaSPP/SPPLs* in different tissues. (**A**) Expression patterns of *BnaSPP/SPPLs* from the BnIR, scale bar is Log2 (TPM)-normalized expression, and blue denotes low expression and red denotes strong expression; the axes indicate *BnaSPP/SPPLs* the and phylogenetic tree. (**B**) RT-qPCR validation of *BnaSPP5*, *BnaSPP6*, *BnaSPPL4-3*, *BnaSPPL3-4*, and *BnaSPPL4-4* in different tissues.

The expression profiles of 26 genes in various tissues or organs were examined based on BnIR in order to examine the role of *BnaSPP/SPPLs* in plant growth and development (Supplementary Table S4). Most genes were expressed throughout the plant; however, the tissue expression selectivity of each gene varied. The expression profiles of *BnaSPP1-BnaSPP6* were remarkably similar, with high expression throughout the plant, notably in 14–30 daf seeds. Among them, *BnaSPPL3-1*, *BnaSPPL3-2*, and *BnaSPPL1-5* were mainly expressed in 14–32 daf seeds, 10–14 daf siliques, and roots; *BnaSPPL1-6* showed very low expression or was almost not expressed; *BnaSPPL3-5* was mainly expressed in 10–16 daf siliques and 14–24 daf seeds; and *BnaSPPL5-2* and *BnaSPPL5-1* were specifically and highly expressed in sepals and pollen, respectively, indicating that they are related to flower development (Figure 6A). To validate the BnIR expression database, RNA was collected from different tissues of ZS11, including the roots, stems, cotyledons, young leaves, mature leaves, buds, pollen, and siliques (Supplementary Table S5). Five *BnaSPP/SPPLs* were chosen at random for RT-qPCR analysis. The results showed that the expression levels were significantly higher in mature leaves, buds, and siliques than in other tissues; among them, the highest expression level was found in pollen, and these expression levels were largely compatible with the transcriptome data (Figure 6B). In summary, *BnaSPP/SPPLs* play crucial roles in the growth and development of *B. napus*.

### 2.5. BnaSPPL4 Regulates Male Fertility in Rapeseed

**Figure 7:**
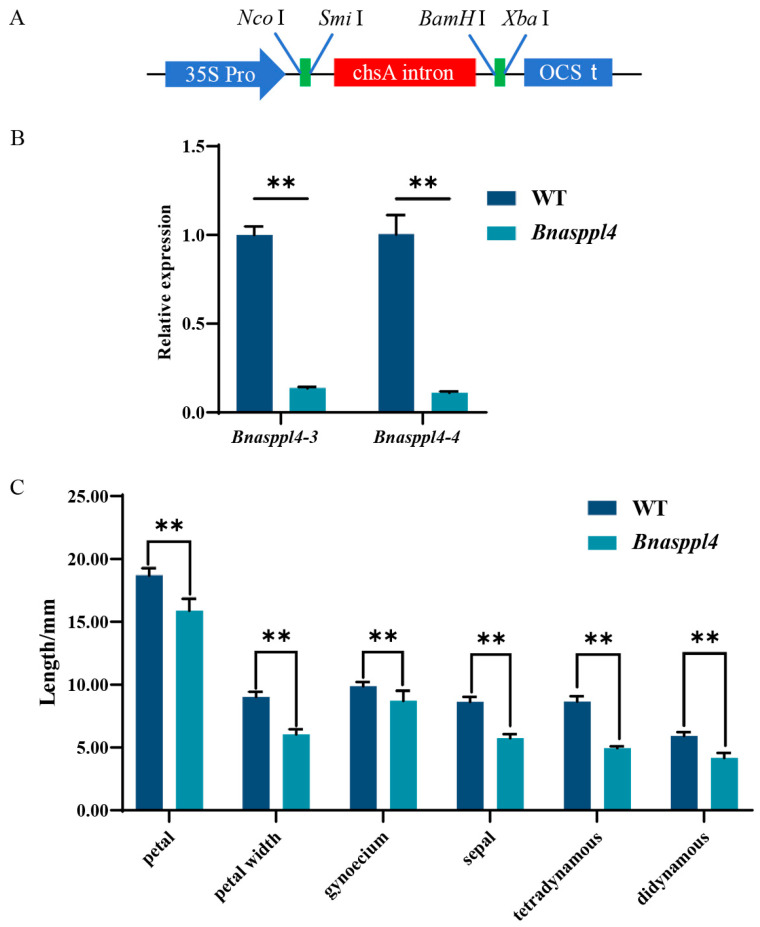
Construction of vector and detection of gene transcription level in transgenic rapeseed. (**A**) Schematic diagram of RNAi vector construction, and the expression vector was pFGC5941. (**B**) Gene transcription level of transgenic rapeseed; data are shown as mean ± SE (*n* = 3). (**C**) Statistical analysis of the size of petals, sepals, stamens, and pistil of transgenic rapeseed and WT; data are shown as mean ± SE (*n* = 20). The symbols ** indicate a statistically significant deviation from WT at *p* < 0.01 probability levels.

**Figure 8:**
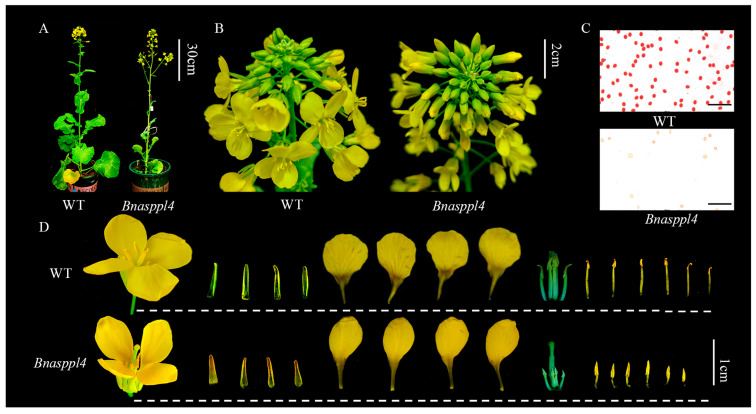
Photographic observation of transgenic rapeseed and wild-type (WT) phenotype: (**A**) phenotype of whole-plant rapeseed; (**B**) inflorescence of rapeseed; (**C**) the pollen was colored with carmine acetate stain solution (bars for 200 µm); (**D**) the petals, sepals, stamens, and pistil of rapeseed were photographed and observed.

Compared with the WT, the gene expression levels of *Bnasppl4-3* and *Bnasppl4-4* were significantly decreased in *Bnasppl4* (Supplementary Table S6). The petals were significantly shorter and narrower, the sepals were shorter, and the stamens were significantly degraded and their length was half that of the WT. The pollen was colored using a carmine acetate stain solution, which showed a significant decrease in pollen activity and the presence of deformed pollen (Figure 7 and Figure 8).

**Figure 9:**
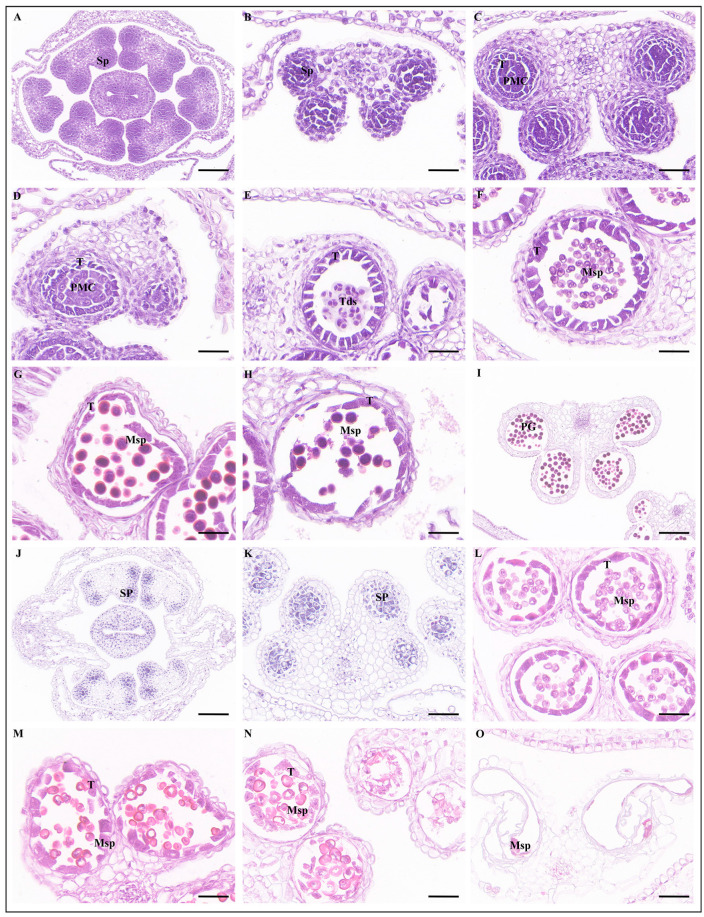
Paraffin sectioning cross-sections: fertile (**A**–**I**) and sterile (**J**–**O**); (**A**,**B**,**J**,**K**) sporogenous cell stage; (**C**) pollen mother cell stage; (**D**) premeiosis stage; (**E**) tetrad stage; (**F**,**L**–**N**) late uninucleate microspore stage; (**G**,**H**) bicellular pollen stage. Sp, sporogenous; PMC, pollen mother cell; Tds, tetrads; Msp, microspores; PG, pollen grain; T, tapetum. (**A**,**J**) bars for 100 µm, magnifications for 200. (**B**–**I**,**K**–**O**) bars for 50 µm, magnifications for 400.

At the sporogenous cell stage of WT anthers, sporogenous cells were located in the center of the anther chamber and closely arranged (Figure 9A). With the development of anthers, the space between sporogenous cells gradually increased (Figure 9B). At the pollen mother cell stage, the tapetal cells were closely arranged in the innermost layer of the anther cell wall (Figure 9C). Tetrads were formed at the end of meiotic period, microspores were released, and the tapetal cells began to degrade (Figure 9D,E). In the late uninucleate stage, the microspores were triangular and had thickened pollen walls (Figure 9F). Vacuoles were then formed in the cytoplasm of the microspore. After asymmetric mitosis, the microspores developed into a bicellular pollen with a vegetative cell and a generative cell, and the tapetum continued to deteriorate, providing nutrients for pollen development (Figure 9G). After undergoing mitosis again, the bicellular pollen became a trinucleated pollen with one vegetative cell and two generative cells, and the pollen was circular (Figure 9H). At the mature stage of anthers, the tapetum completely disappeared and the pollen sac dehisced, releasing pollen grains (Figure 9I).

A cross-section of the Bnasppl4 anthers showed that six stamens had abnormal development and some did not have pollen sacs (Figure 9J,K). Microspore mother cells developed and formed tetrads through meiosis before further developing into mononuclear microspores (Figure 9L). At the late uninucleate stage, microspore development was abnormal, bicellular pollen could not be formed without undergoing mitosis, and the vacuoles in the cytoplasm of microspores gradually expanded, leading to vacuolization of the cytoplasm of uninucleate microspores. At the same time, the nucleus gradually disappeared, the cell wall gradually ruptured of microspores, and tapetal cells were completely degraded and disappeared (Figure 9M,N). Finally, the pollen sac cavity was left behind (Figure 9O).

## 3. Discussion

In this study, we successfully identified 26 *BnaSPP/SPPL* genes and divided them into three groups, which were unevenly distributed on 17 chromosomes and had highly similar genetic structural features in each group. Inter-chromosomal interactions between *BnaSPP/SPPLs* and an analysis of the syntenic relationships between species indicated that the *SPP/SPPLs* of *B. napus* were mainly derived from genome evolution with *B. oleracea* and *B. rapa*, and the evolution mode was purifying selection, forming tandem duplicates on chromosome ChrA09 during the WGD process. *BnaSPP/SPPLs* are widely expressed in various tissues, while *BnaSPPL4* is mainly expressed in mature leaves, buds, siliques, and anthers, and thus may play an important role in the development of siliques and anthers. Therefore, the RNAi expression vector of *pFGC5941::BnaSPPL4* was constructed and transgenic rapeseeds were obtained. It was found that the mutant whose *Bnasppl4-3* and *Bnasppl4-4* expression levels were significantly reduced had obvious male sterility characteristics: the petals, sepals, and stamen were significantly smaller and shorter, and the pollen activity was significantly decreased. Paraffin sections of mutant anthers showed that microspore development stagnated at the late uninucleate microspore stage and eventually formed empty pollen sacs during pollen development. 

SPP/SPPLs are I-CLiPs with catalytic activity [1,19,20,25]. However, a variety of critical non-catalytic physiological functions have been found that cannot be ignored, for example, the TMD-binding ability of inactive rhomboid pseudoproteases [35]. As the initial member of the *SPP/SPPL* family, SPP is mainly located in the endoplasmic reticulum and plasma membrane in plants and animals [7,19,25,36], and its degradation activity is conserved [37,38], which may be related to signal peptide processing in plants [39]. SPPL2 differs from other members of the *SPP/SPPL* family in that it contains an N-terminal signal sequence and a complex glycosylated intracellular/extracellular domain of the nine-TMD segment [11]. SPPL2a and SPPL2b are synthesized in the endoplasmic reticulum and can up-regulate IL-12 transcription when mediated by TNF-α. Recent studies have shown that SPPL2c can cleave TA proteins [29,30] and is only specifically expressed in testes [29,40]. SPPL3 is localized in the Golgi apparatus and widely expressed in humans [40,41]; it is involved in the regulation of the immune system and tumor defense, and is listed as one of the genes that increase the risk of breast cancer [42]. 

SPP/SPPL have only been identified in *Arabidopsis thaliana* [10] and rice [28]. *ATSPP* and its five homologous *ATSPPs* have been successfully identified, among which ATSPP is located in the endoplasmic reticulum, and *ATSPPL1* and *ATSPPL2* are located in the endosome [10]. Arabidopsis pollen develops into tritenucleate pollen grains, and the male germ unit (MGU) is composed of one vegetative cell and two sperm cells [43]. ATSPP is mainly highly expressed in the vegetative organ. The heterozygote of T-DNA insertion lines has MGU defects, resulting in interrupted pollen germination [10]. *MtSPP* is highly expressed in the roots of Medicago truncatula, and it is co-regulated by the NCR (nodule-specific cysteine-rich) gene during nodule formation to produce conserved rich oligopeptides in nodule cells [44].

In angiosperms, anther and pollen development plays a crucial role in the production of male gametophytes [45], and is regulated by a large number of genes [46]. In the CMS lines of *B. napus*, there are a large number of sterility genes, such as orf222, orf288, and orf346 [47,48,49], which produce mitochondrial proteins and cause mitochondrial dysfunction and eventually male sterility. A variety of TGMS genes have recently been successfully identified, such as SP2S [50], H50S [51], and 373S [52]. In addition, a large number of sterile genes have been identified, such as *BnaC. MAGL8a* caused abnormal development of the tapetum layer and led to male infertility [53]. In *Brassica juncea*, transgenic plants expressing Arachis diogoi cysteine protease (AdCP) under the TA-29 promoter showed abnormal development of the tapetum layer [54]. After editing *BnaRFL11* with CRISPR/Cas9, the pollen grains of the homozygous mutant were completely degraded, and the type of anther abortion was similar to that of nap-CMS. The expression levels of the Orf222, Orf139, Ap3, and Nad5c genes in the mutant were significantly up-regulated [55]. Although numerous sterility genes have been identified, many of the mechanisms underlying CMS and GMS remain unclear. TE5A is a recently discovered heat-sensitive male sterile line derived from a mutant inbred line of TEA. It exhibits normal pollination and seed setting at low temperatures but becomes completely sterile when the temperature exceeds 20 °C due to abnormal meiosis development and homologous chromosome pairing [56]. The 373S sterile line is a temperature-sensitive sterile line with spontaneous mutation, and its candidate sterile gene is *Bnms^T1^*, which causes male sterility through abnormal degeneration of the tapete layer [52]. The Huiyou 50s variety was developed through the hybridization of 7401 with common varieties, followed by continuous self-pollination. Huiyou 50S is classified as a high-temperature-sensitive sterile line, with its fertility being controlled by recessive nuclear genes. Cytological observations reveal the occurrence of irregular tetrad cells during meiosis, the degeneration of microspore protoplasts in the late mononuclear stage, and eventually a complete disappearance of pollen grains [57].

## 4. Materials and Methods

### 4.1. Identification of SPP/SPPL Family Members

The genome sequence and gene annotation database of ‘Zhongshuang11’ (ZS11) were obtained from the *B. napus* genome database BnPIR (http://cbi.hzau.edu.cn/bnapus/, accessed on 25 April 2023) [58]. The reference sequences utilized were *ATSPP/SPPLs*, and the E-value < e^−10^ was set for comparison using the BLASTP program [59]. Hidden Markov Model (HMM) profiles of the Peptidase_A22B domain (PF04258) were obtained from the Pfam protein database (Pfam: Home page (xfam.org), accessed on 25 April 2023) [60], and the Simple HMM Search in the TBtools (v 1.133) software was used to identify members. The longest transcripts of each member at the intersection of the two identification results were selected, and the domains were manually searched using the NCBI (https://www.ncbi.nlm.nih.gov/, accessed on 25 April 2023) and SMART (https://smart.embl.de/, accessed on 25 April 2023) websites to identify family members. Expasy [61] (https://www.expasy.org/, accessed on 25 April 2023) and WoLF PSORT (https://wolfpsort.hgc.jp/, accessed on 25 April 2023) [62] were used to forecast the physicochemical properties and subcellular localization. The genome sequence, gene annotation information, and amino acid sequence of *B. oleracea* and *B. rapa* were downloaded from the Ensembl Plants database [63] (https://plants.ensembl.org/index.html, accessed on 25 April 2023) and identified using the methods described above. Finally, *SPP/SPPL* family members were identified in *A. thaliana*, *B. oleracea*, *B. napus*, and *B. rapa*.

### 4.2. Phylogenetic Analysis of Members of the SPP/SPPL Family

The amino acid sequences of the family members were extracted using TBtools, followed by MUSCLE comparison using MEGA 11.0 software [64]. After cropping and aligning the comparison results, a phylogenetic tree was constructed using the maximum-likelihood (ML) method with 1000 bootstraps. The results of the phylogenetic analysis were further visualized and improved using the Evolview 2.0 [65] website (https://evolgenius.info//evolview-v2/, accessed on 25 April 2023) for an aesthetically pleasing and informative presentation. The conserved motifs of the *BnaSPP/SPPLs* and *ATSPP/SPPLs* were analyzed using the MEME website (https://meme-suite.org/meme/, accessed on 25 April 2023) [66]. Gene annotation information was used to investigate the gene structures of *SPP/SPPLs*. In addition, the gene structure domains were predicted using the Batch CD Search tool on the NCBI website (https://www.ncbi.nlm.nih.gov/guide/domains-structures/, accessed on 25 April 2023), and conserved motifs and gene structures were visualized using TBtools [67].

### 4.3. Chromosomal Localization and Covariance Analysis of BnaSPP/SPPLs

Chromosomal positional information of the *BnaSPP/SPPLs* was extracted from the gene annotation of the ZS11. Paralog pairs were obtained through comparison using the MCScanX tool in TBtools [67], with the parameters set to E-value < e^−10^ and number of hits ≤ 5. Subsequently, information about the positions of *BnaSPP/SPPLs* on chromosomes, tandem repeats, and chromosome segment duplications was visualized using Advance Circle in TBtools. The Ka/Ks values of paralog pairs were calculated in order to evaluate the evolutionary mode.

### 4.4. Expression Pattern Analysis of BnaSPP/SPPLs

HeatMap in TBtools was utilized to show the expression patterns, which were obtained from the *B. napus* multi-omics information resource (BnIR) [68]. In order to verify the gene expression data of BnIR, the Promega RNA Extraction Kit (Promega, Beijing, China) was utilized to extract RNA from the roots, stems, cotyledons, young leaves, mature leaves, 4 mm buds, and siliques of ZS11. A ThermoFisher Scientific Reverse Transcription Kit (ThermoFisher Scientific, Shanghai, China) was used to obtain cDNA from 1 μg RNA. Specific primers were designed using Primer-BLAST (https://www.ncbi.nlm.nih.gov/tools/primer-blast, accessed on 25 April 2023) (Supplementary Table S1). RT-qPCR was performed using SYBR Green fluorescence in the CFX96TM Real-Time System, with the program set as follows: 50 °C for 2 min and 95 °C for 10 min, followed by 40 cycles of 95 °C for 15 s and 60 °C for 60 s. The 2^−∆∆Ct^ method was utilized to determine relative expression with *BnaActin2* serving as the reference gene [69]. At the same time, the relative expression in leaf 1 was normalized to the value 1.0. Data were analyzed using ordinary one-way ANOVA in GraphPad Prism 9.0.0.

### 4.5. Plasmid Construction and Genetic Transformation of Rapeseed 

SPPL4 contains four homologous genes in ZS11—*BnaA09G0696100ZS*, *BnaA09G0717600ZS*, *BnaA10G0003600ZS*, and *BnaC05G0005500ZS*—these are referred to as *Bnasppl4-1*, *Bnasppl4-2*, *Bnasppl4-3*, and *Bnasppl4-4*, respectively. A 100 bp conserved sequence of *BnaSPPL4* was selected and primers were designed using SnapGene. SPPL4-RNAi-F: 5′-TGCTCTAGAGCACCATGGGCAGCTATATTCGCCGAAACG-3, where the underlined TCTAGA and CCATGG are the recognition sites of restriction enzymes *Xba* I and *Nco* I; SPPL4-RNAi-R: 5′-CGCGGATCCGCGATTTAAATGCAGACCAATAAGAAGCACAAAGA-3′, where the underlined GGATCC and ATTTAAAT are the recognition sites of restriction enzymes *BamH* I and *Smi* I. The target sequence was assembled into pFGC5941 through enzyme digestion and ligation, and the RNAi vector *pFGC5941::BnaSPPL4* was successfully constructed. This vector was transferred into Agrobacterium tumefaciens strain GV3101 and then transferred into ZS11 through the genetic transformation of rapeseed. At the same time, 35S-F and SPPL4-RNAi-R were used to verify transgenic rapeseed. Rapeseed strains and vectors were provided by the Key Laboratory of Hunan Provincial on Crop Epigenetic Regulation and Development, Hunan Agricultural University, Changsha, China. 

### 4.6. Phenotypic and Paraffin Section Observations 

At the full flowering stage, the petals, sepals, pistils, and stamens of mutant and WT flowers were photographed with a camera (Canon EOS 90D, Canon Inc., Tokyo, Japan). A total of 20 flowers were collected and the length of each flower apparatus was measured and analyzed statistically. In order to analyze the specific period of male sterility of the mutants, hematoxylin–eosin staining and paraffin sections of anthers at different developmental stages were conducted. At 10 am, three anthers were collected from both WT and *Bnasppl4* and broken into slides. Then, 1–2 drops of 1% carmine acetate stain solution were added, and the anther samples were placed under a light microscope (Leica DM3000, Wetzlar, Germany) for observation. Three fields of view were selected for each anther to be photographed under. 

To further study the anther development process of sterile mutants, different-sized buds (0–1 mm, 1–2 mm, 2–3 mm, 3–4 mm, 4–5 mm, 5–6 mm, and >6mm) were taken. The buds were vacuum infiltrated for 30-60 min with 100 mL FAA (50% alcohol 89 mL, 38% formaldehyde 5 mL, glacial acetic acid 6 mL), and fixed at room temperature for 24 h until they completely sank to the bottom of the bottle. A conventional paraffin sectioning method was adopted, and gradient dehydration was carried out with 75%, 85%, 90%, and 95% ethanol. Transparent xylene was applied to the samples. Then, they were paraffin-impregnated and -embedded to section thicknesses of 4 µm, before being stained with hematoxylin–eosin and sealed with neutral gum. Finally, an upright optical microscope (NIKON ECLIPSE E100, Tokyo, Japan) and an imaging system (NIKON DS-U3, Tokyo, Japan) were used for image acquisition and analysis. The paraffin section scheme, image photographing and scanning equipment, and reagents and apparatus involved in the anther sectioning experiment were all provided by Wuhan Servicebio Technology Co., Ltd. (https://www.servicebio.cn/, accessed on 25 September 2023).

## 5. Conclusions

In this study, we identified 26 members of the *SPP/SPPL* family in rapeseed and systematically analyzed their gene structures, domains, and expression profiles. We obtained *Bnasppl4* mutants through RNAi and genetic transformation that showed obvious male sterility phenotypes. Paraffin sectioning showed that the male sterility of *Bnasppl4* was caused by an abnormal development of microspores at the late uninucleate stage. Our study confirmed the role of *BnaSPPL4* in the development of rapeseed pollen, and lays a foundation for further research on the molecular mechanism of pollen development. It also provides valuable germplasm resources for creating new rapeseed sterility lines.

## Figures and Tables

**Table 1:** The physical and chemical properties of *SPP/SPPL* family proteins in *B. napus*.

Gene ID	Gene Name	Location	Size (aa)	MW (Da)	PI	Instability Index	Aliphatic Index	GRAVY	Subcellular Localization
*BnaA01T0045500ZS*	*BnaSPPL1-1*	ChrA01:2433664:2434985	372	41018.71	8.04	42.55	119.01	0.677	Plasma membrane
*BnaA03T0536700ZS*	*BnaSPPL1-2*	ChrA03:30318596:30319895	369	40534.99	8.07	42.34	115.45	0.661	Plasma membrane
*BnaA04T0115800ZS*	*BnaSPPL1-3*	ChrA04:13250245:13250983	162	17983.62	8.44	33.35	120.8	0.722	Plasma membrane
*BnaA06T0148900ZS*	*BnaSPPL1-4*	ChrA06:9010737:9011720	181	20081.97	8.9	35.97	112.43	0.556	Vacuole
*BnaC01T0051800ZS*	*BnaSPPL1-5*	ChrC01:2869609:2870914	372	40985.54	8.38	44.11	118.47	0.69	Plasma membrane
*BnaC03T0754800ZS*	*BnaSPPL1-6*	ChrC03:73142539:73142914	86	9306.36	8.71	26.01	136.05	1.052	Extracell
*BnaC07T0513500ZS*	*BnaSPPL1-7*	ChrC07:58840667:58841964	369	40612.19	8	41.16	113.85	0.656	Plasma membrane
*BnaA09T0148500ZS*	*BnaSPPL2-1*	ChrA09:9059052:9062997	539	59646.09	5.83	30.28	117.37	0.465	Plasma membrane
*BnaC09T0161800ZS*	*BnaSPPL2-2*	ChrC09:12535426:12539195	523	58342.52	6.47	32.66	117.25	0.422	Plasma membrane
*BnaA04T0273700ZS*	*BnaSPPL3-1*	ChrA04:24217292:24217905	106	11527.72	5.15	37.04	113.96	0.632	Plasma membrane
*BnaA04T0273800ZS*	*BnaSPPL3-2*	ChrA04:24217912:24219967	316	35405.88	8.77	43.34	106.71	0.447	Plasma membrane
*BnaA05T0036100ZS*	*BnaSPPL3-3*	ChrA05:1990653:1993716	541	59360.25	5.02	45.91	109.74	0.45	Plasma membrane
*BnaC04T0039100ZS*	*BnaSPPL3-4*	ChrC04:3621314:3624386	541	59330.22	4.96	46.27	109.04	0.455	Plasma membrane
*BnaC04T0591400ZS*	*BnaSPPL3-5*	ChrC04:69507056:69509666	447	49674.56	9.02	48.29	106.82	0.301	Plasma membrane
*BnaA09T0696100ZS*	*BnaSPPL4-1*	ChrA09:64489204:64492337	539	59719.51	7.82	39.9	115.12	0.472	Plasma membrane
*BnaA09T0717600ZS*	*BnaSPPL4-2*	ChrA09:65650441:65653480	563	62510.74	8.04	41.33	113.68	0.448	Plasma membrane
*BnaA10T0003600ZS*	*BnaSPPL4-3*	ChrA10:210105:213788	540	59798.44	6.41	35.29	114.37	0.505	Plasma membrane
*BnaC05T0005500ZS*	*BnaSPPL4-4*	ChrC05:446704:450260	540	59710.27	6.21	35.34	114.74	0.507	Plasma membrane
*BnaA08T0309700ZS*	*BnaSPPL5-1*	ChrA08:27801075:27803833	542	59207.14	5.43	36.34	104.34	0.399	Plasma membrane
*BnaC08T0020500ZS*	*BnaSPPL5-2*	ChrC08:1651410:1654438	540	58824.06	5.81	38.24	105.43	0.463	Plasma membrane
*BnaA02T0326000ZS*	*BnaSPP1*	ChrA02:29422039:29424283	343	37885.76	8.65	34.41	109.42	0.613	Plasma membrane
*BnaA06T0399700ZS*	*BnaSPP2*	ChrA06:45757268:45759541	335	37165.11	8.82	32	112.03	0.655	Plasma membrane
*BnaA09T0223100ZS*	*BnaSPP3*	ChrA09:15794170:15796398	336	37266.19	8.81	34.5	112.53	0.654	Plasma membrane
*BnaC02T0439400ZS*	*BnaSPP4*	ChrC02:54167767:54170281	343	37915.78	8.65	36.31	109.13	0.6	Plasma membrane
*BnaC07T0282000ZS*	*BnaSPP5*	ChrC07:42592380:42594688	335	37139.07	8.82	32.31	112.33	0.665	Plasma membrane
*BnaC09T0259800ZS*	*BnaSPP6*	ChrC09:24544910:24547180	337	37553.64	9.15	36.28	113.06	0.659	Plasma membrane

## Data Availability

Data is contained within the article or Supplementary Materials.

## Supplementary Materials

The following supporting information can be downloaded at: https://www.mdpi.com/article/10.3390/ijms25073936/s1.
